# Facilitators and Barriers Perceived by German Teachers Considering Basic Life Support Education in School—A Qualitative Study

**DOI:** 10.3390/ejihpe14060117

**Published:** 2024-06-18

**Authors:** Rico Dumcke, Claas Wegner, Sabine Wingen, Niels Rahe-Meyer

**Affiliations:** 1 Faculty of Biology/Biology Didcactics (OZHB), Bielefeld University, 33615 Bielefeld, Germany; claas.wegner@uni-bielefeld.de; 2 Department of Anaesthesiology and Intensive Care Medicine, Faculty of Medicine and University Hospital Cologne, University of Cologne, 50937 Cologne, Germany; sabine.wingen@uk-koeln.de; 3 German Resuscitation Council, 89070 Ulm, Germany; 4 FOM University of Applied Sciences, 50678 Cologne, Germany; 5 Clinic for Anaesthesiology and Intensive Care Medicine, Franziskus Hospital Bielefeld, 33615 Bielefeld, Germany; niels.rahe-meyer@franziskus.de

**Keywords:** implementation, school development, BLS, first aid, barriers

## Abstract

This qualitative study aims to analyse the personal qualification, attitudes and the pedagogical concepts of German teachers as experts in their profession regarding basic life support (BLS) education in secondary schools. Thirteen (*n* = 13) secondary school teachers participated in semi-structured expert interviews and were interviewed for at least 20 to 60 min regarding BLS student education. Interviews were semi-structured with guiding questions addressing (1) personal experience, (2) teacher qualification for BLS and (3) implementation factors (e.g., personal, material and organisational). Audio-recorded interviews were analysed by content analysis, generating a coding system. School teachers provided a heterogeneous view on implementation-related processes in BLS education. Many teachers were educated in first aid, acknowledge its importance, but had no experience in teaching BLS. They want to assure being competent for teaching BLS and need tailored trainings, materials, pedagogical information and the incorporation into the curriculum. Also, the management of time constraints, unwilling colleagues, or young students being overwhelmed were commonly mentioned considerations. Concluding, teachers reported to be willing to teach BLS but a stepwise implementation framework incorporating practice-oriented qualification and educational goals is missing.

## 1. Introduction

Böttiger et al. [1] (p. 87) wrote, in their KIDS SAVE LIVES statement on school teachers’ education and qualification in bystander resuscitation, that “Teachers can easily be educated…” and they “already know how to teach, and our program adds the specifics of CPR education”. The “program” they referred to is a four-hour course developed by the German Resuscitation Council (GRC) [2] to transfer the required competencies for BLS education to regular school teachers.

But even if this and other training concepts are available, broad implementation seems to be delayed in Germany [3] and in other European countries, e.g., Denmark or Belgium [4,5]. What are the reasons that these countries are stuck in limited, regional pilot projects?

Presumably, there is no longer any argument against the broad evidence of effective BLS teaching by school teachers or effective learning from the lower secondary level (10–12 years) onwards [6,7,8,9,10]. Rather, we need to assume a discrepancy between theory and practice, caused by several educational tasks that schools have to comply with [11]. As Zinckernagel and colleagues [12] illustrated in their qualitative study, a substantial inhibitory concern of teachers is the assessment of BLS and cardiac arrest (CA) as a “matter of life and death”, as crucial medical knowledge that they have not been trained for. This personal uncertainty, if cumulating with other factors in schools, federal and local curricula, or insufficient infrastructure, may lead to a limited capacity for the development of local BLS programs at schools. Since several quantitative investigations showed general categories of inhibitory issues—equipment, teacher training or no obligatory guidelines, for instance [11,13,14]—qualitative in-depth investigations of the teachers’ motives, concerns, thoughts and recommendations are rare.

Therefore, this study aims to investigate, in more detail, what are the potential pathways for further implementation and what are the obstacles when attempting sustainable BLS education? According to the research principle of design-based research (DBR) [15], this approach directly includes practitioners of the field. By analysing guided interviews, implications prior to or during implementation can be derived and may lead to future solutions.

## 2. Background: Educational Systems and Innovations

### 2.1. Strategies to Transfer Innovations

School development research has shown that transfer processes of innovative concepts or strategies in schools are not as easily adopted as in other economic environments [16]. The autonomy and self-organisation of each school were reported as main reasons for slow transfer and rejected innovative processes [17,18]. Teachers are predominantly self-reliable for their pedagogical acting. Because schools are public institutions, pressure and competition to foster change are lowered. Holtappels [18] (pp. 279–280) reported, on schools’ application of innovations, that several conditions make prescribed, standardised programs unlikely to succeed due to resistance and adverse understanding of aims. The mentioned conditions apply even more to BLS education, because regularly it is no part of the academic training of teachers. Therefore, a flexible and consensus-oriented strategy is likely to be important (known as: normative–educative strategy [18]) to avoid a separation of the level of conception (e.g., initiatives and educational policy) and the level of application (e.g., schools and teachers), as well as in order to integrate and consider the needs and ideas of practitioners [11,16] (p. 200).

### 2.2. What Influences Transfer?

Innovation, as a process, describes the time until changes or new practices become routine (implementation) [19] (p. 3). Scientific research has the function of analysing factors that facilitate or inhibit innovational change [16] (p. 197). Gräsel [20] (pp. 10–13) described four dimensions of characteristics that are relevant to implementation’s success or failure. Frist, the innovation itself is a relevant factor. The more necessary, advantageous and flexible (e.g., stepwise) and the less complex the change is, the more likely the change will be accepted. Then, the development and management of each school affects the organisational change: the administration has to support its teachers to be motivated to adopt innovations and to collaborate to prepare solutions for daily practice. Third, the transfer support is an essential factor for successful implementation. This includes a reliable framework for financial and material issues, special teacher trainings and networking structures to spread information [20] (pp. 10–12). Finally, the characteristics of the teaching personnel determine their acceptance, attitudes, motivation and concerns about the progress of change [21].

### 2.3. Experts of Practice: The Role of Change Agents

To consider the needs and ideas of teachers as agents of change, the application of participative strategies and/or design research as seen growth recently [15,20,22]: educational settings are designed and revised in collaboration with researchers and practitioners in reiterated cycles until a usable solution is established [23].

For changing a system, a group of persons is needed who drive the change. Rogers [24] describes these motivated and convinced people in his Diffusion of Innovation Theory as “early adopters”, who are very important multipliers for the majority (approximately 70%). They explain the innovation’s content and its benefits, they organise materials and they develop school-specific adoptions. In a sense, these adopters—here teachers—are coordinators of change and establish communication with more resistant or hesitant colleagues. However, according to Rogers [24], the stage of dissemination depends on each individual: the knowledge of the innovation is followed by persuasion of its need or benefit. Then, a decision is made about the implementation or rejection [18]. This first stage of loose commitment is marked by non-use or individual attempts to change practice. Usually, these first arrangements are of rather mechanical or superficial use. Institutional acceptance and routine develop when collaborative structures are established [18]. As an indicator of success, Coburn [25] specifies:

- spread (i.e., quantitative dissemination),
- depth (shift of persuasion and pedagogical attitudes),
- sustainability and shift in reform ownership (i.e., identification), both supporting long-term intrinsic motivation [19,25].

The characteristics of transfer and the qualitative factors of implementation transfer can be visualised in a modified utilisation of learning opportunities model, according to Lipowsky and Rzejak [26].

**Figure 1:**
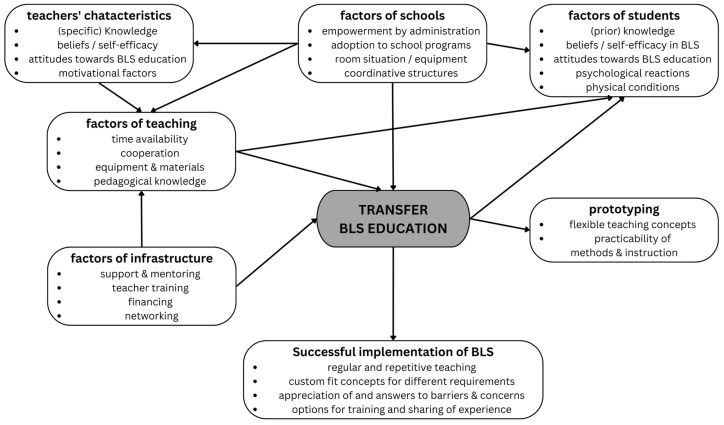
Transfer processes visualised by the utilisation of learning opportunities model for innovations in BLS education. Adopted and modified, see Lipowsky and Rzejak [26] (p. 380).

Figure 1 describes the interactions between individual factors of teachers, factors of teaching and schools, factors of infrastructure (i.e., transfer support) and factors of students for the implementation process of BLS education in secondary schools.

## 3. Research Objectives

In line with the identified challenges when implementing new approaches in schools [16,27,28], which have also been reported recently for the introduction of BLS lessons [11,12,13,29], a closer examination behind the curtains seems expedient. As usual in design research, and as postulated by Coburn [25], a qualitative analysis and dialogue with practitioners is the method-of-choice to differentiate the content of existing quantitative findings and add in-depth information.

Therefore, the aim of this paper is the elaboration and synthesis of motives, concerns, thoughts and recommendations of teachers with and without experience in BLS teaching at secondary schools by conducting semi-standardised interviews [30]. The interview manual was designed according to the following three areas of research interest.

Since individual characteristics, such as perceived utility and practical relevance, increase the willingness to adopt change [20,31,32], research question one asked participants:

**Research Question 1:** *What attitudes and values do teachers assign to BLS education?*

The teachers’ own general and pedagogical competence perception as well as medical self-efficacy were reported to be another crucial variable in BLS investigations with teachers [12,33]. Thoughts and cognitive patterns were addressed by research question two during interviews to understand latent concerns:

**Research Question 2:** *How do teachers evaluate the required competencies to implement and/or teach BLS to students?*

Implementation processes are dependent on highly diverse and specialised educational systems, the German federal system, for instance, and local organisational frameworks [11,34]. Subsequently, international challenges and barriers [4,14,35] may be similar, but should be cautiously compared. Therefore, the examination of details regarding addressed age, curriculum matching, teaching structures and special (qualifying) needs was addressed by research question three:

**Research Question 3:** *How can teachers imagine the process of implementation and what do they need to fulfil this (e.g., financially, materially, in terms of professionalisation, etc.)?*

## 4. Study Design and Methods

This study is part of the initiative and research project “SAVING LIVES MEETS SCHOOLS” (Translated into English from the German original “LEBEN RETTEN MACHT SCHULE”), which started in 2018 as a design-based research project (DBR). In line with the nature of DBR, it follows a multi-step, multi-method approach “to design and develop an intervention (such as programs, teaching–learning strategies and materials, products and systems) as a solution to a complex educational problem […]” [36] (p. 15). The research process consists of three repetitive cycles: (1) preliminary research to identify problems and set a theoretical framework, (2) a development phase to plan, test and revise design concepts or interventions and (3) an assessment to evaluate the effectiveness of the solution (cf. [23,36]). This qualitative research is part of the problem identification regarding the implementation of designs in the school environment. In a technical sense, the interviews thus belong to the first preliminary research phase. We actually used them to re-assess in-school conditions again and in more detail in the design phase between the prototype design and the transfer design test in the field. This decision was made in order to be able to integrate these findings into prototyping.

The interviews aimed at a convenient sample of teachers recruited based on availability and willingness. Teachers who (a) were employed at a secondary school and (2) were located in the region of Westphalia (Germany) could be invited to participate. Individual experience with BLS teaching was not an exclusion criterion.

The interviews were mainly performed and recorded online between February 2021 and March 2022 due to flexibility using the ZOOM Client (https://community.zoom.com/, accessed on 1 February 2021; San José, CA, USA). In some cases, single interviews were performed at workplaces. In both cases, interviews were additionally audio-recorded using a stereo-voice recorder (Olympus LS-series, Hamburg, Germany). Minimum duration was set at 20 min, and maximum at 60 min. Participation was voluntary and participants signed a consent form for recording, analysing and publishing the anonymised results.

### 4.1. Semi-Structured Interview

The methodology of expert interviews in particular considers the design of the interview situation by structuring the process and the positioning of roles involved [30] (p. 670). We used a semi-structured interview with guiding questions. Guidelines were previously defined and systematically applied to organise the conversation [30] (p. 670). The semi-structured format was chosen in order to address the already known general issues from literature research (e.g., [11,13,37]).

The main guiding questions are presented in Table 1. The six questions were designed according to (a) questions left unsolved by findings from quantitative evidence [11] and (b) the utilisation of the learning opportunities model (Figure 1) for innovations in BLS education, and they were designed as open questions or prompts (“question—answer” or “narrative prompt—narration” script). To maintain flow, detailed questions or keywords were presented on the second level of the manual (reported in Supplementary Materials S1). Prior to the interview, years of service, taught subjects and gender were registered.

### 4.2. Qualitative Analysis

The qualitative analysis was conducted according to Mayring [38] as a summarising content analysis. Each interview was interpreted as a recording unit (thirteen cases).

For the recorded audio data, transcription was performed using *f4transkript* (https://www.audiotranskription.de/en/f4transkript/, accessed on 1 February 2021; dr. dresing & pehl GmbH, Marburg, Germany) following Kuckartz’s [39] transcription rules. For each interview, units of coding (i.e., phrases and statements) were inductively assigned to and structured using a theme-based coding frame (i.e., categories), consisting of main and subordinate codes [40].

Main categories were deductively designed by the structure of the guiding manual and the theoretical and empirical basis of the area of research. The subordinate categorial system was defined inductively after paraphrasing relevant interview sections. The categorial system was tested with 25% of the material using *f4analyse* (https://www.audiotranskription.de/en/f4analyse/, accessed on 1 February 2021; dr. dresing & pehl GmbH, Marburg, Germany), and categories were revised and specified, before a final coding with the whole interview material was performed (for categories and rules of coding, see Supplementary Materials S2). Interviews were coded by two pedagogically and methodologically trained raters (one of them R.D.), and intercoder reliability was determined for ensuring quality. Moreover, big tent criteria for qualitative research [41] and the COREQ statement [42] were followed (see Supplementary Materials S3).

## 5. Results

### 5.1. Participants

The interviews addressed pedagogical professionals with experience in the field (i.e., in a school setting) and varying knowledge regarding BLS education. This differing experience was addressed to assess the perspectives and ideas of both experienced and inexperienced teachers. The sample is reported in Table 2. On average, the interviewed teachers were in service for 15.3 years (±11.95), with the shortest experience being 10 weeks and the longest being 28 years. Sixty-nine percent of the participating teachers were female. The mean duration was 36 min. Eighty-five percent of the teachers taught biology (*n* = 11). The second subject was distributed heterogeneously (Table 2).

### 5.2. Coding System

The emerging category framework for analysis is presented in Table 3 and the coding manual in Supplementary Materials S2. The three main categories of the interview guideline, experience, professional development and implementation, were subdivided into ten categories in total (Table 3), of which implementation covered six and the other categories covered two subcodes each. The results were presented as paraphrased and underlined with examples as appropriate.

### 5.3. Intercoder Reliability

According to quality criteria of qualitative research [43], Cohen’s Kappa [44,45] was calculated for 23.1% of the interviews, containing 120 codes (22.2%). The analysed interviews were independently rated by two coding persons with experience in conducting semi-structured interviews. Cohen’s Kappa resulted in κ = 0.68, which can be considered as substantial or good agreement (κ = 0.60–0.79) according to Landis and Koch [46] and Altman [47].

### 5.4. Teachers’ Relation towards BLS and Teaching BLS

Besides one person, who was on duty as a paramedic and experienced both successful and unsuccessful CPR attempts, most of the interviewed teachers had no genuine experience with BLS (coh. 3, resp. 2, par. 3). All other teachers stated similarly:

I haven’t had to perform CPR to anyone yet. (coh. 2, resp. 1, par. 4)

I have not been involved in this (i.e., bystander resuscitation) yet, not even in my private life. (coh. 5, resp. 3, par. 3)

However, about half of the respondents were trained in first aid, as they are obliged to participate in first aid courses every two years and renew their certificate:

As a physical education teacher (I am) also required to keep this first aid training up to date, so to say, every two years. (coh. 2, resp. 3, par. 2)

Another four participants reported first aid certification, but not as regularly as the others—sometimes connected to the need for more regularly offered first aid courses by their employer (coh. 2, resp. 2, par. 14).

Two of the participating teachers reported prior experience in health professions, as an Emergency Medical Technician (EMT; “Rettungssanitäter”) and in nursing. Finally, three participants were certified as first aid instructors at their schools in order to be allowed to run a “school first aid service”, a service at German schools organised by trained teachers in case of sickness or other medical incidents until the ambulance services arrive.

With respect to the experiences in teaching BLS to students as an instructor on a regular basis, teachers were ambivalent in their opinion. On the one hand, most of them dared to teach this issue, but on the other, many had no or rare opportunity to do so. Only one person believed the presence of a more proficient expert would be welcomed:

In biology class, I know at least that in grade six one topic is the cardiovascular system. However, I myself have not yet taught it. (coh. 4, resp. 3, par. 2)

Three interviewed teachers successfully taught BLS or established programs at their schools (e.g., coh. 1, resp. 1, par. 41).

Regarding the value BLS adds to the students’ education, or the opposite, if it had a limited importance in relation to all the relevant educational goals, every teacher welcomed the introduction of first aid competencies into school education in general:

Basically, I would like it to be part of the school education. (coh. 1, resp. 1, par. 69)

Yes! I think it’s a good idea to teach that across the entire population. And the school, of course, is the best place to do it. (coh. 5, resp. 1, par. 35)

If you have the long-term vision that there are many people in the public who are reasonably competent with bystander resuscitation, who also have the confidence to do it in an emergency situation. Then (.) it is important to practice regularly with the students in class. (coh. 5, resp. 1, par. 25)

On closer inquiry, some concerns became evident. For many responding teachers, anxiety and a feeling of being stuck or overwhelmed was predominantly connected to their conception of BLS, and this reaction is what they expected from other citizens if they were confronted with a cardiac arrest situation. This negative feeling was also transferred to students’ attitudes and their role as BLS instructors. One person even doubted that students benefit from regular BLS education and described thatyoung people should not be exposed to this hard decision-making and responsibility. A call for help, this would be enough until a legal age.

…I don’t know if I would incorporate it into the lessons. So, I see pros and cons. With the younger students, no way! (coh. 1, resp. 3, par. 18)

This assessment contradicts the view of some other teachers who expressed that the primary achievement of BLS education should be the relaxed handling of first aid and the reduction of fears for life-long learning:

But I think it’s much more important (…) from as young as possible, to have a relaxed approach, so that they have already done something like this before, (…) feel confident enough to be able to do it. I think it’s more a matter of an attitude that you can create at an early stage (…) (coh. 4, resp. 1, par. 22)

So, the sooner you start, the better. And you don’t have to start in grade 9, you can do it earlier. (coh. 2, resp. 1, par. 22)

### 5.5. The Role of Competencies and Skills for Teaching BLS

The participants assessed their own competencies in teaching BLS to students very differently. A couple of interviewees stated to be quite positive and self-efficacious in conducting BLS lessons. The main reasons provided were prior teaching experience, prior medical profession and continuous re-certification:

But I can do that because I have acquired a couple of experiences. (coh. 1, resp. 2, par. 5)

However, the majority set a certain condition under which they believe they are able to implement BLS trainings: having specialised content training.

In this regard, some voted for self-studying BLS extensively. Another group focused on the doing: they want “some practice” or at least a qualifying instruction in using BLS manikins. One teacher clearly expressed having too little personal practice in BLS and would refuse to teach it—she explained she was afraid of lacking the expected authenticity:

But with the level of training I have right now in this field, I wouldn’t be comfortable teaching it to my students. (…) and then it would certainly be fair if my students say, “Yeah, if you can’t even do that right, how are we supposed to learn it?” (coh. 5, resp. 1, par. 33)

Two individuals summarised that, as a teacher, one needs advanced knowledge to sufficiently educate students on this medical issue, answer questions correctly and be persuasive (i.e., not only mastering the algorithm itself):

Well, I think to get confident in your acting, you need a deepened knowledge and not just a glimpse. (coh. 1, resp. 2, par. 5)

When asked about good conditions for becoming a professional educator for cardiac arrest and BLS, 11 of the 13 participating teachers at least recommended, some even demanded, short trainings that focus on hands-on practice and/or classroom-relevant activities:

And I want to practice and, to try out on the manikins, so that I can teach that appropriately. (…) and a little medical background you should know as well: How does the cardiovascular system work? Why do we have to keep pumping? (coh. 2, resp. 2, par. 18)

…as an absolute necessity, however, I clearly believe in advanced training courses to ensure that people are properly trained in this respect. (coh. 4, resp. 3, par. 18)

As a personal motivation, they referred to the assessment of BLS being a crucial issue and the need to pass on things correctly (coh. 4, resp. 3, par. 22). Another participant highlighted especially the pedagogical contents that should be provided:

Yes, you could offer further training for teachers (…) That you get informed for educational practice: what kind of task assignments can be used, what kind of group exercises can be done. What details you have to pay attention to. It is, yes/the devil usually is in the details. (coh. 4, resp. 2, par. 23; 25)

However, all teachers, even those participants who favoured self-learning instead of trainings, wanted to be supplied with ready, attractive teaching materials and adequate information adopted to their educational setting or type of school, e.g.,

The training, where you get something like: “This is a series of lessons, you can use it, it’s been tested, you can use it like this”. (coh. 3, resp. 1, par. 82)

Some, however, subsequently argued it would be good to receive flexible teaching materials with “open spaces” (coh. 1, resp. 2, par. 35) or suggested creating their own materials for their lessons but having a contact person for pedagogical feedback on it.

Finally, one person pointed out a potential lack of appreciation for the teachers’ job and said that respect must be paid to colleagues who spend their (extra) time and it has to be decided about the value BLS is assigned among the whole curriculum:

That’s always a story with teachers, because it’s always taken for granted that they simply do things on the side in addition to their teaching duties. (coh. 1, resp. 1, par. 45)

### 5.6. Factors Influencing Implementation of BLS Education

#### 5.6.1. Staff

Professionals! Definitely, professionals! (coh 1, resp. 1, par. 24)

According to the question of the most appropriate instructor, all participants mentioned or even preferred external professionals (medical or aid organisations’ staff): predominant reasons were the impact of uniforms, of narrative authenticity and experiences, or simply variety (coh. 1 resp. 1, 2; coh. 2, resp. 1; coh. 4, resp. 1, 2).

However, at least one person recognised that engaging third parties causes time and financial burdens when it comes to broad availability (coh. 5, resp. 3, par. 22f.). In this context, there are also respondents who considered all or some teachers to be suitable trainers. Mainly, natural science/biology (because of their life science knowledge) and physical education teachers (because of their first aid certification) were cited:

Basically, anyone with a proper training could teach it. (coh. 2, resp. 2, par. 16)

For practical reasons, I can imagine it most likely to happen with physical education teachers. (coh. 5, resp. 1, par. 29)

#### 5.6.2. Equipment

Besides the obvious availability of a “series of these manikins, […]” (coh. 4, resp. 1, par. 38), which nearly all participants demanded, several other conditions for teaching BLS were expressed:

- Copy templates that you can already use. Pictures, a PowerPoint… (coh. 1, resp. 1, par. 49)
- A short script with a tutorial. (coh. 1, resp. 2, par. 21)
- I would like to have a manual (…) Which topics have to be done in which order…? (coh. 4, resp. 3, par. 34)
- Provided teaching materials at different levels (…) which can be used for all types of schools, i.e., appropriately differentiated. (coh. 1, resp. 3, par. 36; 48)
- A handout, with information about statistics. (coh. 3, resp. 1, par. 80)
- Informative material, maybe also worksheets, practical tasks, maybe something like that. (coh. 4, resp. 3, par. 34)
- Ready-made teaching models. (coh. 5, resp. 1, par. 45)
- Ready-made course plan, simply designed. (ibid., par. 47)

For most teachers, the main focus here was on saving time by having ready-to-use materials, which at the same time would provide them with content-related, medical certainty. They highlighted that the materials should be differentiated according to complexity, (co-)developed by didactic experts and provide small-step explanations, (e.g., frequent questions) for successful implementation:

Well, the good material is decisive for the colleagues to really implement it reasonably, otherwise they don’t have any interest. (coh. 1, resp. 2, par. 27)

#### 5.6.3. Organisation

Responses on organisational efforts can be divided into three issues, which were named by all the participants (directly or indirectly):

1. *Room capacity and availability*: Many teachers criticised that normal classrooms do not provide enough space for appropriate training with manikins. For the preparation (e.g., moving heavy tables), the daily time is missing. Therefore, good planning of rooms must be prepared and reviewed (sports hall, assembly hall, etc.) at the school level.

A very simple practical reason: if now the students are supposed to do their BLS exercise with their partner, how is that supposed to work in a class of 30? (coh. 5, resp. 1, par. 29)

2. *Shortage of time*: Some teachers assumed about 3–4 lessons (e.g., coh. 4, resp. 2, par. 40) or 6–8 lessons within four weeks (coh. 4, sep. 3, par. 48) for BLS education. Predominantly, participants felt they “never have enough time” (coh. 5, resp. 1, par. 27) and that “the school year [is] very tight with time” (coh. 1, resp. 1, par. 43) for regular responsibilities, and even less so for additional BLS lessons. It was not clear what content should be shortened without regulations.
3. *Unique framework regulations*, which subsidise the process of implementation in each school, as illustrated by one participant (coh. 5, resp. 1, par. 67): discussing the issue of BLS and relevant grades in teams, including the principal, and initiating a pilot program followed by decision-making at a school conference. Finally, the school program or an internal curriculum must be updated and communicated to all of the colleagues and responsibilities have to be assigned. Some teachers predicted problems regarding cooperation—agreements between different subjects, etc.—or the integration at the classroom level by single teachers:

(laughs) cooperation with colleagues can always be very difficult. (coh. 2, resp. 1, par. 68)

Of course there are the internal school curricula, but what is really done is another story. (coh. 4, resp. 1, par. 34)

#### 5.6.4. Obligatory Thoughts

Among the participating teachers, a general denial of additional workload without any form of compensation was perceptible (e.g., coh. 5, resp. 1, par. 37). One person highlighted this, providing some examples:

[I mean,] that the colleagues get the credit accordingly. Either through hours of compensation or through overtime payment or opportunities for job promotion. (coh. 1, resp. 1, par. 45)

The acceptance of investing more time seemed to be limited, with participants specifying about 3–4 h. But a whole day, much less during off time on Saturdays, was not tolerable (coh. 1, resp. 2, par. 61) for composing materials, searching for information or undergoing training (coh. 3, resp. 1, par. 52).

Some teachers pointed to the present curriculum provided by the ministries of education and said that without a legal foundation, which affords the teachers a “special timebox” within which they have to deal with this BLS content (coh. 4, resp. 1, par. 34), implementation could fail:

[It is] basically just difficult to insert that “just like that” into the lesson if you don’t frame it appropriately in the core curriculum beforehand. (coh. 2, resp. 3, par. 14)

Clearly needs an obligation. (…) (Smiling) I’m not that idealistic. (coh. 5, resp. 3, par. 105)

Two participants added their impression that, if a legal basis is not provided along with appropriate acknowledgement of extra engagement, many colleagues would resist or withdraw from participation, because other subject-matter content or exams are more urgent (coh. 1, resp. 1, par. 61; coh. 1, resp. 2, par. 25; coh. 5, resp. 1, par. 27).

And you can already see how tightly packed the contents are in the different grades, and there are certainly many colleagues who complain directly […]. (coh. 4, resp. 3, par. 46)

### 5.6.5. Emotional Concerns

Addressing the emotional charge of an issue such as BLS, some of the interviewed teachers expressed concerns regarding the maturity of young students and their role in providing first aid. Whereas one person strictly warned against giving children a responsibility that some adults do not have, another person reflected on recognising potential bad experiences with relatives, and on being cautious and prepared:

They are children, and adults should take responsibility for children. (coh. 1, resp. 3, par. 22)

As soon as you have to deal with the subject of death and so on, then you have to keep an eye out. (coh. 2 resp. 1, par. 58)

One perspective was to establish a well-being atmosphere, and at least within younger ages, to avoid the “thinking about death” and instead focus on clear guidelines and hands-on routines (coh. 2, resp. 3, par. 30). However, that does not mean that emotionality diminishes within higher grades, especially considering puberty in 14/15-year-old students, their position towards the body, fear of contact with the other gender, etc. (coh. 2, resp. 3, par. 24).

Another critical issue was the performance and physical ability of the students, which was questioned by some interviewees for students in grades 5 and 6:

I’m just not sure, grade 6, to what extent they’re already able to do that there. (coh. 1, resp. 2, par. 23)

But on the other hand, positive assessments were also made, especially when classifying the primary goal of BLS teaching: to create the right mindset at an early stage where even sixth graders are not too young (coh. 4, resp. 1, par. 22; 40).

### 5.6.6. Implementation and Subjects

Asking teachers for an appropriate frame or the placement within subject-matter teaching, the variety of answers was wide and heterogeneous. However, according to this sample, three main opportunities were presented:

1. The organisation during biology class. The main reasons for this were the closeness to some of the curricular contents, such as circulation, human heart cycle, respiration and circulatory diseases in humans, etc., in grade 6. Another reason was the expertise of the subject teachers in human biology (cf. coh. 1, resp. 1; 2; 3; coh. 2, resp. 1; 2; coh. 3, resp. 1; coh. 4, resp. 2; 3).

Biology sixth grade: the human biology. (cf. coh. 1, resp. 3, par. 38)

2. Secondly, many teachers favoured physical education (P.E.) lessons for implementation. In addition to the fact that physical education teachers are believed to have high first aid skills, practical considerations were mentioned: Indoor sports facilities offer space without moving tables (coh 5, resp. 1, par. 73) and there are usually no exams to be conducted in this subject. However, some teachers would rather welcome a cross-curricular conjunction of these two subjects for a repetitive BLS curriculum, although they noted at the same time that cooperation is difficult (cf. chapter 5.6.3).

In physical education, you also deal with “health education”, so you could make another excursion (…). You could repeat the basic content from the sixth grade [in biology]. That besides is a little more flexible in which grade you have to repeat that [BLS] again. (coh. 4, resp. 2, par. 29)

So this combination of subjects [biology/P.E.] is ideal for this, I believe. (coh. 1, resp. 1 par. 35)

3. The third, more common (and often used) but more detached proposal, was an integration of BLS content into project teaching: as part of a “health awareness day”, with BLS included, or as a fixed “theme week” for several grades at the same time (coh. 1, resp. 2, par. 25; coh. 4, resp. 1, par. 34). The main advantage of this approach seems to be the availability of extra staff or the easy invitation of external organisations or BLS experts when the regular schedule is paused.

Finally, two other successful concepts were presented: the conduction of hands-on BLS sessions during spontaneous replacement lessons (if no other tasks were assigned; coh. 5, resp. 2, par. 17) or the implementation into so-called “community lessons” held by the class teachers, where usually social issues or general problems are discussed (coh. 5, resp. 1, par. 67).

However, a general beginning of BLS education—despite the difficulty and complexity of its content—was endorsed from grade 6, i.e., age 11–12, with emphasis on repetition:

And I think with each grade, as I mentioned, from the sixth grade on, they’ve done the heart structure. Then you can build on that. (coh. 3, resp. 1., par. 60)

But that maybe you say sixth and eighth or again in upper grades [note: grades 11–13]. (coh. 4, resp. 3, par. 40)

## 6. Conclusions and Implications

**Figure 2:**
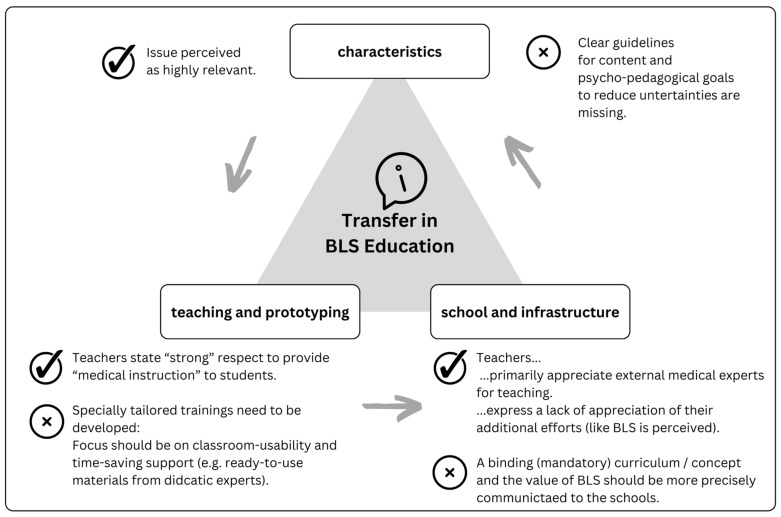
Key findings with reference to the utilisation of learning opportunities model (see white boxes).

The present qualitative investigation provided important insights into the perspective of German teachers regarding a regular or mandatory BLS teaching during secondary school. Key findings are visualised in Figure 2.

### 6.1. Perceived Relevance Is Paired with Uncertainties

In general, the interviewed teachers assigned a high level of relevance for the society and social learning of BLS education and were mostly trained in first aid. Several quantitative investigations have underlined these findings [4,48,49]. Nevertheless, two opposing positions can be identified: on the one hand, those who reject the “burden of responsibility” for children in early grades, and on the other hand—the majority—who consider a particularly early introduction of BLS content to be beneficial. Besides recommendations for early introduction [50], remaining doubts must be addressed and argued with all experts and practitioners.

Recommendation: For BLS instruction on a broad basis, pedagogical guidelines must be discussed and determined that strike a compromise between early introduction in the school career and the avoidance of stressful components, such as “death”, “penalty” and “obligation”. It needs to be made clear which activities should be performed at specific ages to achieve the defined learning objectives.

### 6.2. Need for Qualifying and Time-Saving Resources

So far, some quantitative observational studies have shown that available training and materials are limiting factors in the implementation of BLS-related activities (in summary, cf. [4,11,13]). This investigation indicated a correlation between both good qualification programs and the usability of teaching materials to enhance the willingness to actually provide BLS education to schoolchildren. Some teachers confirmed exactly what Zinckernagel and colleagues [12] reported: namely, that there is strong respect for not being sufficiently skilled to teach the details of cardiac arrest, BLS and upcoming medical questions in front of students. The principle “teachers need to know more than students” seems to apply here, in particular. Teachers need not only technical knowledge, but also the know-how for planning lessons, new methods and emotional support—according to Iserbyt et al. [35] and Madou et al. [51], “specialised content knowledge (SCK)”. Another important message is that teachers struggle with additional time resources. To make up for this and, above all, to balance for their own competence gaps, they demand materials prepared or reviewed by didactic experts, which also consider differences in learning groups in grades 6–13.

Recommendation: When establishing qualification programs for teachers to conduct BLS at their schools, the focus should not be only on performing BLS (as Greif et al. [52] propose, for instance), but also on pedagogical core skills, such as:

- providing instructions and feedback constructively,
- creating (hands-on, creative, reflective, etc.) tasks,
- balancing methods (role play, simulation, discussion, etc.).

Teachers should be provided with the “tools of practice”, which is not only presenting a video but also a structured task to work with. This way, it becomes usable as a whole “classroom idea”—maybe for biology or P.E., as suggested. However, the flexibility and modular structure is highly feasible.

### 6.3. A Matter of Appreciation

Surprisingly, all participants in this study said that an external professional was welcomed to perform the job. One reason for this was certainly the authenticity that comes from experience in the position. However, there was a hidden desire to reduce the workload: one less thing to worry about and do oneself. Understandably, therefore, in addition to adequate funding for exercise materials, such as manikins, a mark of recognition was demanded for that special engagement [34]. Recognition here means:

- Strong backing by the school management [14],
- Reduction of the weekly time schedule,
- Remuneration for coordination and responsibility,

Lastly, and probably most importantly, a decision of where—instead of what—the issue finds its mandatory allocation within the curriculum is needed [53].

Recommendation: Ministers and others responsible in educational administration should decide that BLS (e.g., along with general health education) becomes mandatory to give schools and teachers a binding framework that they base their concept on.

As a final note, it is important to understand that every student should be educated in basic activities: check—call—compress. This theory needs communication to achieve awareness and understanding. This is not to be mistaken with a complete first aid course, nor is this aim achieved if a facultative after school club takes place. Young generations at large should become lifesavers in the future.

## Figures and Tables

**Table 1:** First level (main) guiding questions/prompts of the interview manual. For each question, factors (keywords) of the utilisation of learning opportunities model (Figure 1) were assigned.

Semi-Structured Interview: Guiding Questions (See Also Supplementary Materials S1)		Keywords (cf. Figure 1)
1.1	Why don’t you describe to me what private or school experiences you personally have with bystander resuscitation?	Teachers’ characteristics
1.2	How do you value non-professional bystander resuscitation… (a) personally? (b) as a teaching objective?	Teachers’ characteristics Factors of teaching Factors of students
2.1	Who do you think should teach bystander resuscitation in schools?	Factors of teaching Factors of schools
2.2	Would you be confident in teaching this yourself, and would you attach conditions to it to make it work?	Teachers’ characteristics Factors of infrastructure Factors of schools
3.1	How can you imagine an implementation at your school so that all secondary students receive instruction in bystander resuscitation on a regular basis?	Factors of infrastructure Factors of schools Factors of students Prototyping
3.2	(If not already mentioned): Can you also imagine the issue being included in subject-matter classes or being taught in certain grades?	Factors of teaching Factors of students Prototyping

**Table 2:** Interview characteristics of participating secondary school teachers.

No.	ID ^1^	Gender	Professional Experience (Years)	Subjects/Expertise	Duration (min)
1	coh. 1, resp. 1	female	23	Biology/Phys. Edu.	33
2	coh. 1, resp. 2	male	21	Biology/Music	30
3	coh. 1, resp. 3	female	20	Biology/Maths	43
4	coh. 2, resp. 1	female	12	Biology/Chemistry	26
5	coh. 2, resp. 2	female	3	Biology/Maths	19
6	coh. 2, resp. 3	male	1	Biology/Phys. Edu.	29
7	coh. 3, resp. 1	female	20	Biology/Religion	59
8	coh. 4, resp. 1	male	26	Biology/Latin	23
9	coh. 4, resp. 1	male	0.8	Biology/Phys. Edu.	23
10	coh. 4, resp. 3	female	0.2	Biology/Phys. Edu.	33
11	coh. 5, resp. 1	female	6.5	English/Music	63
12	coh. 5, resp. 2	female	28	Biology/Phys. Edu.	68
13	coh. 5, resp. 3	female	39	German/Religion	52
		MW (SD)	14.2 (12.6)		39.8 (17.1)

^1^ Anonymous labelling: coh. = cohort; resp. = respondent.

**Table 3:** Content coding. Main and subcategories, descriptions and code proportions are presented. Keywords are according to Figure 1.

Categories	Subcategories	Short Description	N_codes_ (%)	Keywords
Personal Experience	Level of training	Statements on the (previous) experiences of the teachers regarding first aid, cardiac arrest and BLS	60 (10.4)	Teachers’ characteristics Factors of infrastructure
	Awareness/emotions/attitude	Expressed attitudes, beliefs and emotional perceptions towards BLS	55 (9.5)	Teachers’ characteristics Factors of students
Professional development	Analysis of competencies	Reported perceptions regarding own skills and knowledge of BLS and teaching BLS	34 (5.9)	Teachers’ characteristics Factors of teaching
	Conditions for personal professionalisation	Statements on individual assumptions to gain more confidence and efficacy as a BLS instructor	79 (13.7)	Teachers’ characteristics Factors of teaching Factors of infrastructure Prototyping
Implementation	Personnel needs/educators	Information on personnel conditions and characteristics of BLS instructors	42 (7.3)	Teachers’ characteristics Factors of infrastructure
	Need for equipment/learning materials	Assessments regarding the required materials, information and technical support for teaching BLS in schools	62 (10.7)	Factors of infrastructure Factors of teaching Prototyping
	Organisational efforts	Perceptions on how a framework for implementing innovative BLS lessons in the context of the school conditions could be organised	92 (15.9)	Factors of infrastructure Factors of schools Factors of teaching Prototyping
	Idealistic/mandatory conditions	Reported opinions about organisational problems connected to extrinsic forces or conditions (colleagues, administration, government, etc.)	43 (7.5)	Teachers’ characteristics Factors of infrastructure Factors of schools
	Emotional–social challenges	Reported considerations on students’ OR teachers’ anxieties/concerns in the context of BLS.	35 (6.1)	Teachers’ characteristics Factors of teaching Factors of students
	Implementation in classroom practice and subject teaching	Ideas regarding implementation of the innovation of BLS according to structures, curricula and practice at the classroom level	75 (13.0)	Factors of teaching Factors of students Prototyping

## Data Availability

The data (interview transcriptions) presented in this study are available on request from the corresponding author due to privacy and data protection regulations.

## Supplementary Materials

The following supporting information can be downloaded at: https://www.mdpi.com/article/10.3390/ejihpe14060117/s1. Supplementary Material S1: Focus questions. Supplementary Material S2: Coding agenda. Supplementary Material S3: COREQ statement [27,31,54,55,56,57].
