# Supplementary material for: Facilitators and Barriers Perceived by German Teachers Considering Basic Life Support Education in School—A Qualitative Study
Source: Eur J Investig Health Psychol Educ. 2024 Jun 18;14(6):1769–85. doi: 10.3390/ejihpe14060117 (PMC11202837; doi:10.3390/ejihpe14060117)
Supplement: Supplementary file 1 [file ejihpe-14-00117-s001.zip › ejihpe-2918703-supplementary.pdf]

Supplementary Material S1

Table S1: Focus questions and prompts of the semi-structured interview (translated into English by the authors). For guiding questions, please refer to the main article text.

**Focus questions according to the main questions (see article text)**	
1.1 Focus questions	• Do you have first aid training?<br>• Have you regularly attended refresher courses?<br>*If yes: how confident do you feel about the training?*<br>• How do you assess your skills?<br>*If not good: what could help you?*<br>• Do you have any personal experiences?<br>*If yes: how did they make you feel?*<br>*If no: do you consider lay resuscitation to be a school-relevant topic?*
1.2 Focus question	Rate the level of importance<br>*(e.g., compared to other topics/tasks)*
2.1 Focus questions	• If professionals: Why professionals?<br>• If colleagues: What better experience do your colleagues have than you (if applicable, related to a subject)?<br>• If others: Who?
2.2 Focus questions	• Why do you (not) have the confidence to do this?<br>*(Professional, pedagogical, or emotional reasons? Prejudices?)*<br>• Are there any preconditions that the school would have to establish? Do you have any examples?<br>• Are there conditions that need to be discussed? Do you have examples?<br>*(e.g., further training, qualification, contents, scope, type, etc.)*<br>*If yes: ask for more details, let examples be given*
3.1 Focus questions	Note: The interviewee may also proceed step-by-step or chronologically.<br>• What organisational processes would have to be conducted with the teaching staff? Assumed problems?<br>• What are—in your opinion—the preparatory steps needed before such an implementation would be possible in practice/in everyday life? Assumed problems?<br>*(e.g., further training, materials, competence requirements, etc.)*<br>If further training: What would be important?<br>If materials: What kind of materials exactly do you mean?<br>How could/must materials be organised?<br>• How would you organise the lessons? Problems?<br>*(Grade level, type, duration, premises?)*
3.2 Focus questions	• If so, where would you attribute/implement that?<br>Do you have an example of how you would incorporate this?<br>• Do you think this suggestion is realistic or do you see problems?<br>*(Time requirements, curriculum structure, cooperation with colleagues, etc.)*

Supplementary Material S2

Table S2: Coding agenda with rules and examples.

Personal experience (PE)			
**Subcategory**	**Definition**	**Coding rules**	**Examples**
**PE1 Level of training**	The teachers' personal previous experience and level of training should be considered during implementation (cf. [1] (p 126)). Here, the (previous) experiences of the teachers regarding first aid, cardiac arrest and resuscitation (e.g., self-performed, witnessed) are described. Experiences may be distinguished into private and professional/educational context, respectively.	Not coded: teachers' professional and didactic competencies<br><br>Mentioned competencies or lack of knowledge and steps of professionalization: **code in PD1.**<br><br>Personal attitudes, beliefs and statements on relevance: **code in PE2**	"I have never had to resuscitate anyone." (coh. 1 resp. 1, par. 3)<br>"I have never had to resuscitate anyone." (coh. 2 resp. 1, par. 4)<br>"I have to do it every two years at school, I'm a first aid provider. We have to refresh the first aid course every two years." (coh. 2, resp. 1, par. 6)<br>"I have, uhm, already done a first aid course several times." (coh. 4, resp. 4, par. 8)
**PE2 Awareness/ Emotions/ Attitude**	Personal attitude towards the issue or innovation is an important factor for the quality and direction of change in implementation processes (cf. [2] (p 225)). Emotions and feelings of teachers are particularly significant, especially for the achievement of teaching goals, but also for the students' interest genesis. Positively experienced feelings influence the perception of success, which is correlated with learning effort (cf. [3] (p 190))	Here, attitudes and emotional perceptions are coded. Mentioned competencies or lack of knowledge and steps of professionalization: **code in PD1.**<br><br>Emotional or social aspects of students' reactions are coded in **IP5**	"You're afraid of causing harm." (coh. 1, resp. 1, par. 17)<br>"Basically, I think it would like it to be part of school education." (coh. 1, resp. 1, par. 69)<br>"Uhm..., I don't know if I would incorporate it into the lessons. So, I see pros and cons. With the younger students, no way!" (coh. 1, resp. 3, par. 18)<br>"But I think it's much more important from an early age, I mean from as young as possible, to have a relaxed approach to it, so that they have already done something like this before, have seen a manikin like this before[...]." (coh. 4, resp. 1, par. 22)<br>"Yes! I think it's a good idea to teach that across the entire population. And the school, of course, is the best place to do it." (coh. 5, resp. 1, par. 35)

Professional development (PD)			
**Subcategory**	**Definition**	**Coding rules**	**Examples**
**PD1 Analysis of competencies**	The competencies of teachers are important factors to drive implementation, which should be adopted and modified according to the skills of the teachers (cf. [2] (pp 225–230)).<br>The main focus here is on the professional competencies of the teachers: how they assess their previous professional (medical, didactic and methodological) competencies, e.g., their knowledge of the cardiovascular system or how to perform chest compressions, how to organize skill trainings, etc. (cf. [4] (p 505)).	Here, previous experience of the teachers (e.g., first aid courses) is not coded: **code in PE1**<br>Further steps towards or conditions for professionalisation related to the issue are not addressed. What teachers need to feel more confident in teaching resuscitation **is coded in PD2**	"Uh, well, I would trust myself to do it, I've already taught it." (coh. 1, resp. 1, par. 41)<br>"Yes, sure, I would definitely need a bit more practice." (coh. 2, resp. 2, par. 6)<br>"But the pressure, which is very important that it reaches the heart at all, I would be extremely unsure about that." (coh. 4, resp. 3., par. 16)<br>"But with the level of training I have in the area, I wouldn't trust myself to teach it to my students." (coh. 5, resp. 1, par. 33)
**PD2 Conditions for personal professionalisation**	An important step in the professionalisation of teachers is the further development of their competencies. This requires the acquisition of new competencies (e.g., in-service training) (cf. [2] (p 230)). Teacher training leads to a change in knowledge and actions, which in turn is the fundament for teaching and students' learning (cf. [5] (341)). Professionalisation is not only a matter of expanding teachers' specific content knowledge (CK, e.g., in the form of first-aid courses), but also of expanding specialised pedagogical (content) knowledge for the given innovation (i.e., didactic training, curriculum planning).	This category only includes the necessary assumptions for the teachers' own professionalisation (type, duration and content of measures)<br>Other factors for the organisation of teaching the innovation issue are **coded in IP1-5**, according to specific content.	"Well, I think to get confident in your acting, you need a deepened knowledge and not just a glimpse." (coh. 1, resp. 2, par. 5).<br>"That's always a story with teachers, because it's always taken for granted that they simply do things on the side in addition to their teaching duties." (coh. 1, resp. 1, par. 45).<br>"I get all the things you need for this [annot.: the lessons] and I get it explained once and then we exercise it in a training course." (coh. 1, resp. 2, par. 15)<br>"Yes, you could offer further training for teachers (…) That you get informed for educational practice: what kind of task assignments can be used, what kind of group exercises can be done. What details you have to pay attention to. It is, yes/ the devil usually is in the details." (coh. 4, resp. 2, par. 23; 25).

Implementation (IP)			
**Subcategory**	**Definition**	**Coding rules**	**Examples**
**IP1 Personnel needs/educators**	During adoption of new innovations, often already employed teachers are appointed to implement new task in their classes, which also causes re-skilling (see above). Schools will rarely try to hire additional staff (cf. [2] (p 230)).<br>However, here, personnel needs are listed in this category: are teachers themselves sufficient or are external personnel required? What are qualifications of instructors/personnel and what are reasons for this view?	Only information on personnel conditions and characteristics were coded<br><br>If conditions regarding the issue were mentioned, such as of materials or organisation, these were **coded in**<br>**IP2 for materials or equipment**<br>**IP3 for organizational efforts**<br>**IP4 for mandatory or limiting conditions**	"Basically, anyone with a proper training could teach it." (coh. 2, resp. 2, par. 16)<br>"For practical reasons, I can imagine it most likely to happen with physical education teachers." (coh. 5, resp. 1, par. 29)<br>"Trained professionals, of course, as this is indeed a sensitive issue." (coh. 2, resp. 3, par. 14)<br>"The natural scientists, especially the biologists, have a certain understanding of the cardiovascular system that other colleagues don't have. So I can understand why teachers like them are brought in." (coh. 4, resp. 2, par. 11)
**IP2 Need for equipment/learning materials**	A key factor in implementation is clarity about learning goals, competencies and concepts for instruction. Handouts and guidelines used for implementation and realization should be well designed [2] (p 222). The complexity of the materials also plays a role, so that as many levels of heterogeneity and local differences as possible are taken into account (cf. ibid. p.223).	Here, only assessments on required materials, information and technical support are coded. Further requirements, are **coded in**<br>**IP1 for personnel**<br>**IP3 for organisational efforts**<br>**IP4 for mandatory or limiting conditions**<br>**IP6 for specific ideas on curriculum od subject-matter teaching**	"Copy templates that you can already use. Pictures, a PowerPoint…" (coh 1, resp. 1, par. 49)<br>"Provided teaching materials at different levels (…) which can be used for all types of schools, i.e. appropriately differentiated." (coh 1, resp. 3, par. 36; 48)<br>"We are already quite well equipped here, we have these manikins that we can use with the students." (coh. 2, resp. 2, par. 24)<br>"A ready-made course plan, simply designed" (coh. 5, resp. 1, par. 47)<br>"A room that is intended for this purpose." (coh. 5, resp. 3, par. 63

Subcategory	Definition	Coding rules	Examples
**IP3 Organisational efforts**	Implementation in educational settings depends—besides others—on three factors: the type of pedagogical innovation, the strategies selected and the organisational framework (cf. [1] (p 48)). Here, only the organisational framework is considered. Process flow, responsibilities, time management and design of the resuscitation-related education are aspects coded here. These aspects also influence the quality of teaching (cf. [6] (168ff.)).	The focus here is on the framework of implementing innovative lessons regarding the innovative subject. When it comes to thematic or subject-matter questions, e.g., for which age groups resuscitation should be taught or what opportunities are given for interdisciplinary collaboration, these are **coded in IP6.** Aspects that refer to mandatory regulations or emotional–social hindrances: **code in IP 5**	"A very simple practical reason: if now the students are supposed to do their BLS exercise with their partner, how is that supposed to work in a class of 30?" (coh. 5, resp. 1, par. 29)<br>"The departmental conference, uh, has to discuss in which grade you want to do it, how you want to do it and uhm... then it will be anchored in the internal school curriculum." (coh. 1, resp. 1, par. 57)<br>"You definitely need time, that's always/, teaching time is always very limited." (coh. 2, resp. 2, par. 24)<br>"Perhaps there will be a pilot class first." (coh. 5, resp 1, par. 67)
**IP4 Idealistic/mandatory conditions**	In the context of implementation, there are various influencing factors that can cause problems in the organisation.<br>Therefore, here, several arguments are included, which are connected to idealistic or mandatory requirements to be met by schools. These include legal provisions or (missing) programs for funding.<br>But also general tasks of educators or professional ethos and attitudes of colleagues are coded, if potential conflicts or benefits are described (cf. [1] (p 217))	Only organisational problems connected to extrinsic forces or conditions are coded here.<br>Other organisational efforts: **code in IP3**<br>Social or emotional considerations: **code in IP5**	"[I mean,] that the colleagues get the credit accordingly. Either through hours of compensation or through overtime payment or opportunities for promotion." (coh. 1, resp. 1, par. 45)<br>"Everyone will argue that their own things are more urgent and more important. The curriculum gets fuller and fuller when we do something like this [annot.: BLS education]." (coh. 1, resp. 2, par. 25)<br>"Oh, the first thing that comes to my mind in terms of organisation is that you get the appropriate material and that you get it funded somehow." (coh. 4, resp. 03, par. 28)

Subcategory	Definition	Coding rules	Examples
**IP5 Emotional–social challenges**	In this context, negative emotions are a particular problem. In particular, anxiety affects students' motivation and lowers their self-efficacy. It also has an influence on social interactions in the classroom (cf. [3] (192f.)). Secondly, also concerns or thought of teachers' or colleagues are collected here, if connected to the action of teaching resuscitation to students.	Here, we will mainly focus on students' OR teachers' anxieties/concerns in the context of resuscitation.<br>If the arguments focus on general (bystander) anxieties of teachers, these **are coded in PE2**<br>If considerations are connected to students or subject-matter ideas, please **code in IP6**	"I'm just not sure, class 6, um, to what extent they are already able to do that." (coh. 1, resp. 1, par. 23)<br>"It has to be fun for the pupils and of course there have to be exercises that they can try out on each other." (coh. 1, resp. 2, par. 47)<br>"[annot.: Regarding children performing CPR:] These are children, and adults should take responsibility for children." (coh. 1, resp. 3, par. 22)<br>"And some colleagues always like to say: 'Nah, that's not my responsibility'." (coh. 5, resp. 1, par. 31)
**IP6 Implementation in classroom-practice and subject-teaching**	In addition to the organisational framework, the integration of the innovation into:<br>• educational policy objectives (e.g., in the general curricula)<br>• or local school curricula of taught subjects or general projects<br>is also important (cf. Goldenbaum, 2012, p.114[1] (p 214)).<br>This category is for local fields and pathways of application (cf. ibid., p.48), e.g., structures of an implementation process, interdisciplinary collaboration, subject-matter, theme based pedagogical ideas, methods or social considerations (age, level of proficiency, etc.)	Here, only the implementation of the innovation according to structures, curricula and practice (at the classroom level) is coded.<br>Further (more general) organisational conditions at the school level are **coded in IP3 or IP4**	"So in biology, that's great, in year 6 we have the lungs, lung function, and you can also treat blood pressure, circulation, everything. And you could really, really tie it in with that." (coh. 1, resp. 1, par. 23)<br>"With the cardiovascular system. In eighth and ninth grade. There's a lot of human biology, for example." (coh. 1, resp. 2, par. 37)<br>"Then it makes the most sense to have a project day so that you can supervise the group with two, three or four teachers." (coh. 1, resp. 3, par. 36)<br>"We then looked to see where it would fit into the curriculum. And we've already included it in the biology and sport curriculum." (coh. 3, resp. 1, par. 37)<br>"In physical education, you also deal with "health education", so you could make another excursion (…). You could repeat the basic content from the sixth grade [in biology]. That besides is a little more flexible in which grade you have to repeat that

	[BLS] again." (coh 4, resp. 2, par. 29)

References

1. Goldenbaum, A. *Innovationsmanagement in Schulen*: *Eine empirische Untersuchung zur Implementation eines Sozialen Lernprogramms;* VS Verlag für Sozialwissenschaften: Wiesbaden, 2012, ISBN 978-3-531-19425-7.
2. Altrichter, H.; Wiesinger, S. Der Beitrag der Innovationsforschung im Bildungswesen zum Implementierungsproblem. In *Psychologie des Wissensmanagements*: *Perspektiven, Theorien und Methoden*; Mandl, H., Reinmann-Rothmeier, G., Eds.; Hogrefe: Göttingen [u.a.], 2004; pp 220–233, ISBN 3840918154.
3. Frenzel, A.C.; Götz, T.; Pekrun, R. Ursachen und Wirkungen von Lehreremotionen: Ein Modell zur reziproken Beeinflussung von Lehrkräften und Klassenmerkmalen. In *Analyse und Bedeutung unterrichtlichen Handelns.*; Gläser-Zikuda, M., Ed.; Waxmann: Münster, 2008; pp 187–209.
4. Baumert, J.; Kunter, M. Stichwort: Professionelle Kompetenz von Lehrkräften: Paralleltitel: Keyword: Professional competencies of teachers. *Zeitschrift für Erziehungswissenschaft* **2006**, *9*, 469–520.
5. Fussangel, K.; Rürup, M.; Gräsel, C. Lehrerfortbildung als Unterstützungssystem. In *Handbuch Neue Steuerung in Schulsystem*; Altrichter, H., Maag Merki, K., Eds.; VS Verlag für Sozialwissenschaften: Wiesbaden, 2019; pp 327–354.
6. Helmke, A. *Unterrichtsqualität und Lehrerprofessionalität*: *Diagnose, Evaluation, und Verbesserung des Unterrichts;* Kallmeyer: Seelze, 2012.

Supplementary Material S3

COREQ Criteria

Table S3: COREQ 32-item checklist with reference to information presented in the article (Tong, Sainsbury, & Craig, 2007, also cf. The Equator Network: https://www.equator-network.org/reporting-guidelines/coreq/)

No.	Item	Guide questions/description	Comment/page
**Domain 1: Research team and reflexivity**			
Personal Characteristics			
1.	Interviewer/facilitator	Which author/s conducted the interview or focus group?	RD and associated teacher students employed at Bielefeld University
2.	Credentials	What were the researcher's credentials? *e.g., PhD, MD*	RD/M.Ed.<br>CW/PhD<br>SW/PhD<br>NRM/PhD, MD
3.	Occupation	What was their occupation at the time of the study?	RD: Bielefeld University<br>CW: Bielefeld University<br>SW: University of Cologne<br>NRM: Fanziskus Hospital Bielefeld
4.	Gender	Was the researcher male or female?	Male
5.	Experience and training	What experience or training did the researcher have?	Three years (RD) and more than 10 years (CW) of experience in qualitative research in the educational sector
Relationship with participants			
6.	Relationship established	Was a relationship established prior to study commencement?	No
7.	Participant knowledge of the interviewer	What did the participants know about the researcher? *e.g., personal goals, reasons for doing the research*	Participants knew about the project goals of the STAYING ALIVE and KIDS SAVE LIVES initiatives and benefits of CPR education in schools
8.	Interviewer characteristics	What characteristics were reported about the interviewer/facilitator? *e.g., bias, assumptions, reasons and interests in the research topic*	None besides the positive attitude towards school-based CPR education
**Domain 2: Study design**			
Theoretical framework			
9.	Methodological orientation and theory	What methodological orientation was stated to underpin the study? *e.g., grounded theory, discourse analysis, ethnography, phenomenology, content analysis*	Content analysis

No.	Item	Guide questions/description	Comment/page
Participant selection			
10.	Sampling	How were participants selected? *e.g., purposive, convenience, consecutive, snowball*	Convenience sample
11.	Method of approach	How were participants approached? *e.g., face-to-face, telephone, mail, email*	Face-to-face, at location, or via online call, if necessary
12.	Sample size	How many participants were in the study?	Thirteen
13.	Non-participation	How many people refused to participate or dropped out? Reasons?	None
Setting			
14.	Setting of data collection	Where was the data collected? *e.g., home, clinic, workplace*	Workplace
15.	Presence of non-participants	Was anyone else present besides the participants and researchers?	No
16.	Description of sample	What are the important characteristics of the sample? *e.g., demographic data, date*	All participants were certified and employed teachers or exam candidates at German secondary schools
Data collection			
17.	Interview guide	Were questions, prompts, guides provided by the authors? Was it pilot tested?	Yes, by using a semi-structured guide. This was tested and revised closely with two associated teachers of the research working group who work at schools
18.	Repeat interviews	Were repeat interviews carried out? If yes, how many?	No
19.	Audio/visual recording	Did the research use audio or visual recording to collect the data?	Audio only, with one backup level
20.	Field notes	Were field notes made during and/or after the interview or focus group?	Yes, but only to postpone arising questions and ask them later during interviews
21.	Duration	What was the duration of the interviews or focus group?	Minimum of 20 and maximum of approximately 60 minutes.
22.	Data saturation	Was data saturation discussed?	No
23.	Transcripts returned	Were transcripts returned to participants for comment and/or correction?	No, due to COVID-19 and (related) time constraints/no interest.

No.	Item	Guide questions/description	Comment/page
**Domain 3: Analysis and findings**			
Data analysis			
24.	Number of data coders	How many data coders coded the data?	Besides RD who coded all interviews, three coders shared second coding.
25.	Description of the coding tree	Did authors provide a description of the coding tree?	Yes, see Supplementary Materials S1 and S2
26.	Derivation of themes	Were themes identified in advance or derived from the data?	Main codes were coded in advance by main categories from the guiding manual, subcodes were generated from the data.
27.	Software	What software, if applicable, was used to manage the data?	F4analyse (dr. dresing&pehl GmbH, Marburg, Germany)
28.	Participant checking	Did participants provide feedback on the findings?	No, due to COVID-19 and (related) time constraints/no interest.
Reporting			
29.	Quotations presented	Were participant quotations presented to illustrate the themes/findings? Was each quotation identified? *e.g., participant number*	Yes, throughout the results section by providing cohort (No.), respondent (No.) and paragraph.
